# Remembering the Legacy of Judi Tuerck

**DOI:** 10.3390/ijns9010001

**Published:** 2022-12-22

**Authors:** Amy Morris, Cheryl Hermerath, Jelili Ojodu, Neil Buist

**Affiliations:** 1 Oregon State Public Health Laboratory, Hillsboro, OR 97124, USA; 2 Association of Public Health Laboratories, Silver Spring, MD 20910, USA; 3 Department of Pediatrics, Oregon Health & Science University, Portland, OR 97239, USA; 4 Department of Medical Genetics, Oregon Health & Science University, Portland, OR 97239, USA

Judith “Judi” Tuerck, RN, MS, one of the true pioneers in the development of newborn screening (NBS), passed away on Saturday, 18 June 2022 (Figure 1). She died at the age of 74 at Oregon Health & Sciences University (OHSU) Tuality Medical Center in Hillsboro, OR, USA. She was born on 15 March 1948 to Patricia Elaine and George Albert Carothers at Jones Hospital, which was to later become the same hospital in which she passed. Judi was the oldest of five children; in 1964, they were all adopted by Elaine’s second husband Paul Fischer.

Education was at the heart of Judi’s core values. She graduated from Lincoln High School in Portland, Oregon, Class of 1966. She went on to get her Bachelor of Science and Nursing from the University of Oregon School of Nursing in 1971. After taking time off to start a family and build her career, she attended the OHSU School of Nursing and earned her Master’s in Public Health in 1986. In 1971, she started her career as a community health nurse for the Washington County Health Department. From 1976 to 1978, she worked as a nursing instructor and the Director of Health Services for Mt. Hood Community College. It was in 1978 that she started the life-changing journey that would become her legacy at OHSU. During her 29-year career at OHSU, she held multiple titles simultaneously, including Program Coordinator for the Metabolic Clinic at OHSU Child Development and Rehabilitation Center (CDRC) as well as Consultant for the NW Regional Newborn Screening (NBS) Program. 

Like most people in a new position, she started out at the metabolic clinic as a rookie. What she lacked in experience she made up for in determination and soon became familiar with the wide array of disorders included in the NBS panel. True to her nature, she did not stop there. It was not long before she expressed the importance of expanding the panel to include even more rare disorders. 

As she settled into her role, she soon discovered holes in the system, presenting the potential that an infant identified with a heritable newborn-screening disorder might not receive the appropriate medical care. Her solution? Education. It was Judi’s idea to create educational programs to be distributed around the region. What started out as a program for six surrounding states became the foundation for the first NBS Practitioner’s Manual that covered all aspects of NBS. This booklet was the first of its kind and was soon copied by other NBS programs in the U.S. and elsewhere. 

After attending a meeting in Portland titled “Full Circle”, covering the filter paper blood spots used as specimens in newborn-screening testing, she realized the importance of the term “full circle” in regards to the roles of different agencies involved in NBS. It was common knowledge that every screening program consisted of a laboratory, follow-up system, and medical treatment center and that these agencies, for the most part, stood alone regarding testing standards and protocols. Judi is the one who recognized that these three components were part of a larger system in which pre-laboratory handling of the specimens, post-result data management, and quality control of each segment were all equally important. When equal value was placed on each system component and standardization was implemented, then the “full circle” would be achieved. 

In addition to the numerous local presentations she gave each year to healthcare professionals in Oregon, during her career she gave scores of national and international educational lectures to change the face of short-term and long-term follow-up care across the globe. She has 62 publications and was on 27 different regional, national, and international committees and still found time to participate in community service to raise awareness for NBS follow-up care. It was because of these contributions that she was presented the Association of Public Health Laboratories (APHL) George Cunningham Visionary Award for service to infants and families in newborn screening in 2007.

Judi was instrumental in shaping newborn-screening systems through her leadership on several national and international committees, including the Clinical Laboratory and Standards Institute (CLSI) Subcommittee on Newborn Screening Guidelines for Sick and Premature Infants; Chair, CLSI Subcommittee on Newborn Screening Guidelines; International Neonatal Screening Society Council; Council of Regional Networks for Genetic Services; National Newborn and Genetics Recourse Center; and APHL Newborn Screening and Genetics in Public Health committees and subcommittees, to name a few.

An avid “cheerleader” for the newborn-screening community and an integral member of the Oregon and Northwest Regional NBS Programs, Judi was instrumental in developing many of the follow-up and educational procedures that are the standard of practice in national and international newborn-screening programs today. 

In 2014, she became the namesake of the APHL Judi Tuerck Newborn Screening Follow-up and Education Award. Recognition for her advocacy, commitment, and efforts on behalf of newborns worldwide will continue to be an important part of the history and legacy of newborn screening. 

Judi retired in 2007 and spent the last 15 years enjoying her life. She continued her love of travelling and went on many out-of-state and international adventures with her family. She had a passion for food and gardening. Her recipes will be shared for generations to come. She was a talented seamstress and taught her granddaughter the joy of quilting. She will be remembered for her generosity, salty wit, and respect for education. Judi’s legendary stories, sense of humor, and dedication will be greatly missed.

She is survived by her daughter, Amy Morris; son-in-law, James Morris; granddaughter, Taylor Morris; granddaughter, Sydney Morris; sister, Jan Watson; and sister, Patricia Shaw. She was preceded in death by her parents, Paul and Elaine Fischer; brother, Mark A. Fischer; and sister, Molly K. Munson.

## Figures and Tables

**Figure 1:**
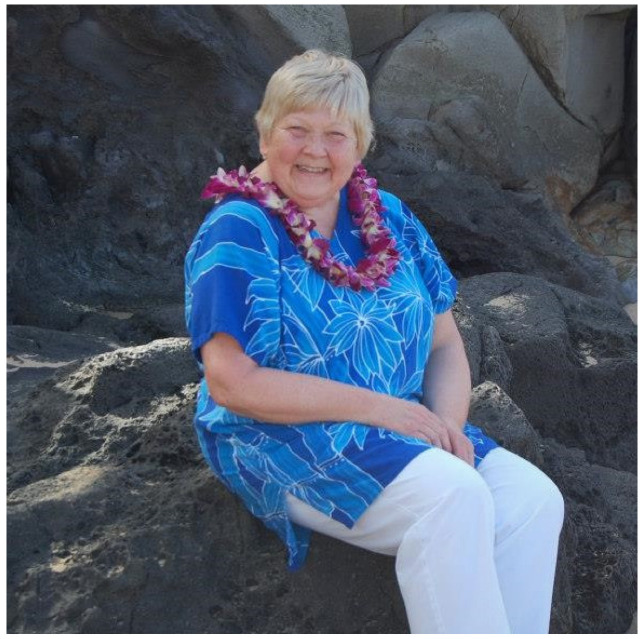
Photo of Judi Tuerck (submitted by her family).

